# Intestinal schistosomiasis among schoolchildren in Sana’a Governorate, Yemen: Prevalence, associated factors and its effect on nutritional status and anemia

**DOI:** 10.1371/journal.pntd.0009757

**Published:** 2021-09-21

**Authors:** Sami Ahmed Al-Haidari, Mohammed A. K. Mahdy, Abdulsalam M. Al-Mekhlafi, Walid M. S. Al Murisi, Ahmed Ali Qaid Thabit, Mohammed Abdullah Al-Amad, Hassan Al-Shamahi, Othman Saeed Bahashwan, Abdulwahed Al Serouri

**Affiliations:** 1 Department of Parasitology, Faculty of Medicine, Sana’a University, Sana’a, Yemen; 2 Diseases Control & Surveillance, Ministry of Public Health and Population, Sana’a, Yemen; 3 Communicable Diseases Department, World Health Organization, Sana’a, Yemen; 4 Field Epidemiology Training Programme, Ministry of Public Health and Population, Sana’a, Yemen; 5 Department of Medical Microbiology, Faculty of Medicine, Sana’a University, Sana’a, Yemen; Ministère de la Santé Publique et de la Lutte contre les Endémies, NIGER

## Abstract

Intestinal schistosomiasis is a neglected tropical disease, causing morbidity and mortality in tropical and subtropical countries. Despite the frequent implementation of mass drug administration with praziquantel, the reinfection with *Schistosoma mansoni* is still common in Yemen. In addition, there is a scarcity of information on the impact of *S*. *mansoni* on nutritional status and anemia among schoolchildren. The present study aimed to determine prevalence and risk factors of intestinal schistosomiasis and investigate its impact on nutritional status and anemia among schoolchildren in Sana’a Governorate, Yemen. It was conducted in 2018 on 445 schoolchildren aged 5–15 years. Biodata, socio-economic, demographic, behavioral and environmental data were collected using a standard questionnaire. *S*. *mansoni* was identified and quantified by microscopic examination of Kato-Katz fecal smear. Hemoglobin concentration and anthropometric measurements were estimated using standard methods. The prevalence of *S*. *mansoni* was higher in Al-Haimah Al-Dakheliah (33.9%) than Bani Mater (1.4%). Household without tap water (Adjusted Odds Ratio (AOR) = 2.9, 95% Confidence interval (CI): 1.12, 7.55, *P* = 0.028) was the independent risk factor of the infection. The prevalence of wasting and stunting was 25.0% (95%CI: 21.2%, 29.2%) and 45.8% (95%CI: 41.2%, 50.5%), respectively. The prevalence of underweight among schoolchildren aged 5–10 years was 27.3% (95%CI: 21.9%, 33.4%). The prevalence of anemia was 31.7% (95%CI: 27.5%, 36.2%) with 0.5%, 21.1% and 10.1% being severe, moderate and mild anemia, respectively. *S*. *mansoni* (AOR = 4.1, 95%CI: 2.16, 7.84, *P* < 0.001) and early adolescence (AOR = 6.8, 95%CI: 4.26, 10.82, *P* < 0.001) were independent predictors of stunting among schoolchildren. The early adolescent schoolchildren (AOR = 3.1, 95%CI: 1.86, 4.97, *P* < 0.001) and children from families with low (AOR = 2.1, 95%CI: 1.01, 4.15, *P* = 0.046) or moderate wealth (AOR = 2.3, 95%CI: 1.11, 4.77, *P* = 0.026) were significantly more wasted.

Early adolescence (AOR = 1.8, 95%CI:1.14, 2.78, *P* = 0.011), female (AOR = 1.6, 95%CI: 1.03, 2.43, *P* = 0.038) and Al-Haimah Al-Dakheliah District (AOR = 3.4, 95%CI: 1.20, 9.55, *P* = 0.021) were independent risk factors for anemia. The study findings indicate highly focal prevalence of schistosomiasis in Sana’a Governorate with a public health significance that varies from low to high risk. Approximately half of schoolchildren were stunted, which was associated with *S*. *mansoni* infection and early adolescence. One quarter of schoolchildren were wasted with early adolescent schoolchildren and children from poor families being at high risk of wasting. Anemia was a moderate public health threat affecting the female and the early adolescent schoolchildren. The study suggests the implementation of control measures to combat schistosomiasis and integrated diseases control programmes to improve the health status of schoolchildren in Sana’a Governorate.

## Author summary

The present study aimed to determine prevalence and risk factors of intestinal schistosomiasis and investigate its impact on nutritional status and anemia among schoolchildren in Sana’a Governorate, Yemen. It was conducted in 2018 on 445 schoolchildren aged 5–15 years. Information was collected using a standard questionnaire. *S*. *mansoni* was identified by microscopic examination of Kato-Katz fecal smear. Hemoglobin concentration and anthropometric measurements were estimated using standard methods. The prevalence of *S*. *mansoni* was highly focal (1.4–33.9%). Schoolchildren living in households without tap water had significantly high infection rate of *S*. *mansoni*. The prevalence of wasting, stunting, underweight and anemia among schoolchildren was 25.0%, 45.8%, 27.3% and 31.7%, respectively. Although *S*. *mansoni* was significantly associated with stunting, no significant association was found between *S*. *mansoni* and wasting, underweight or anemia. The stunting was higher among early adolescent than young schoolchildren. The early adolescent schoolchildren and children from families with low or moderate wealth were significantly more wasted than schoolchildren from families with high wealth. Schoolchildren in the early adolescence, being female or living in Al-Haimah Al-Dakheliah District were at high risk of anemia. The study suggests implementation of control measures to combat schistosomiasis and integrated diseases control programmes to improve the health status of schoolchildren in Sana’a Governorate.

## Introduction

Human schistosomiasis is a neglected tropical disease caused by *Schistosoma* species and occurs mainly in tropical and sub-tropical countries [1]. It causes severe morbidity and mortality with an estimated global burden of 1.4 million disability-adjusted life-years (DALYs)[2]. *Schistosoma* species with high global prevalence include *S*. *haematobium* (urogenital schistosomiasis), *S*. *mansoni* and *S*. *japonicum* (intestinal schistosomiasis)[1]. Intestinal schistosomiasis in schoolchildren compromises growth, physical fitness, cognitive function and educational achievement and causes anemia [3–5].

In Yemen, schistosomiasis has been a public health problem since 1922[6] with a patchy distribution and different infection rates, ranging from 15% to 100% [7–14]. A combined Yemen-WHO project for controlling schistosomiasis was set up in 1973, which estimated that 25% of the population were infected with *S*. *mansoni* and/or *S*. *haematobium* [15,16]. After implementing several campaigns of school-based mass drug administration (MDA) with praziquantel, the prevalence of *S*. *mansoni* at country level dropped to 2.5% with a district-based prevalence ranging from 0.0 to 35.7% [17]. In the nationwide survey conducted in 2017, three years after the previous survey, the prevalence of *S*. *mansoni* increased to 7.4% [18]. Malnutrition and anemia are other threats affecting schoolchildren in Yemen where 59%, 47% and 18% of school-aged children were found stunted, underweight and anemic, respectively [19]. However, there is a paucity of information about the impact of *S*. *mansoni* on the nutritional status and anemia among schoolchildren in Yemen [20]. Thus, the present study aimed to determine prevalence of *S*. *mansoni*, identify factors associated with the infection and its impact on nutritional status and anemia among schoolchildren in rural communities of Sana’a Governorate, Yemen.

## Methods

### Study area, design and subjects

This is a cross-sectional study conducted in the rural areas of Sana’a Governorate, Yemen. Schoolchildren aged 6–15 years were the study population. Children who had taken iron, nutritional supplements or anti-parasitic drugs in the last six months prior to the study were excluded.

### Sample size and sampling strategy

The minimum sample size required for the study was 358 schoolchildren which was calculated by Epi Info | CDC (https://www.cdc.gov/epiinfo/index.html)) using the following parameters: 95% confidence interval, ± 5% precision and the highest recently reported prevalence of *S*. *mansoni* (37%) [17]. However, 445 schoolchildren were enrolled in the study to replace participant for not providing fecal sample. A multistage sampling approach was used for selecting schoolchildren where two districts from rural areas of Sana’a Governorate were randomly selected, followed by random selection of one school from each district. Children from each school were selected by systematic random sampling from the students record until the required sample size was obtained. If a selected child refused to participate or was not eligible, he/she was replaced by the next student in the record. The number of students selected from each school was proportional to the size of the school.

### The study questionnaire

Biodata, socio-economic, demographic, behavioral and environmental data were collected using a pre-designed, structured questionnaire through a face-to-face interview. The questionnaire included questions about durable items, animals and agricultural land owned by households; household’s source of drinking water; sanitation coverage; father and mother education; and the number of household’s members.

### Parasitological investigations

A single fresh fecal sample was collected from each participant in a dry, clean plastic container, labeled with the child’s name and identification number. At the field, a Kato-Katz thick fecal smear was prepared from each fecal sample and the rest of feces were preserved in 10% formalin. The Kato-Katz thick fecal smears were then transported to the Parasitology Laboratory in the Faculty of Medicine and Health Sciences, Sana’a University and examined for *S*. *mansoni* [21]. The intensity of *S*. *mansoni* was classified into light (1–99 EPG), moderate (100–399 EPG) and high intensity (≥ 400 EPG) [22]. The public health significance of the prevalence of *S*. *mansoni* was classified into high risk (≥30%), moderate risk (≥10 and <30%) and low risk (<10%) as suggested by the national control strategy [17].

### Hemoglobin estimation

A single measurement of hemoglobin concentration from each child was conducted using a portable hemoglobin analyzing system HB 301+ (HemoCue1 AB, Angelhome, Sweden) on blood collected by finger-prick following the manufacturer instruction. Children were classified into anemic or non-anemic (Hb ≥ 115 g/l for children aged 5–10 years and Hb ≥ 120 g/l for children aged 11–15 years), and subsequently as mild (Hb = 110–114 g/l for children aged 5–10 years and Hb = 114–119 g/l for children aged 11–15 years), moderate (Hb = 80–109 g/l) and severe anemia (Hb < 80 g/l) after adjusting the hemoglobin measurement for altitude according to WHO reference[23]. The public health significance of anemia prevalence was classified as normal (≤ 4.9%), mild (5.0–19.9%), moderate (20.0–39.9%) and severe (≥ 40%)[23].

### Anthropometric measurements

For anthropometric measurements, standing height of each child was measured to the nearest 0.1 cm using a portable stadiometer (Seca, model 208) and his/her weight was measured to the nearest 0.1 kg using a digital weight scale. The age of each participant was retrieved from the birth certificate or school records. The collected measures were used for calculating height-for-age z-score (HAZ), weight-for-age z-score (WAZ) and BMI-for-age z-score (BAZ) using the WHO AnthroPlus software for the global application of the WHO reference 2007 for 5–19 years[24]. The WHO reference data for WAZ used by the WHO AnthroPlus software were for age ≤ 10 years; therefore, underweight was estimated for children aged 5–10 years old. The nutritional indicators for school-age children were defined as follows:

**Table:** 

**Nutritional indicator**	**Cut-off Z-score**
**Stunting (Height-for-age (HAZ))**	
Stunting	Below– 2 SD of the WHO Growth Standards median for HAZ
Moderate stunting	–2 SD to– 3 SD of the WHO Growth Standards median for HAZ
Severe stunting	Below– 3 SD of the WHO Growth Standards median for HAZ
**BMI (BMI for age (BAZ)**	
Wasting	Below– 2 SD of the WHO Growth Standards median for BAZ
Moderate wasting	–2 SD to– 3 SD of the WHO Growth Standards median for BAZ
Severe wasting	Below– 3 SD of the WHO Growth Standards median for BAZ
**Underweight (Weight-for-age (WAZ))**	
Underweight	Below– 2 SD of the WHO Growth Standards median for WAZ
Moderate underweight	–2 SD to– 3 SD of the WHO Growth Standards median for WAZ
Severe underweight	Below– 3 SD of the WHO Growth Standards median for WAZ

### Statistical analysis

Data were analyzed using IBM SPSS Statistics for Windows, version 23.0 (IBM Corp., Armonk, NY, USA). The wealth indices were determined using the principal component analysis (PCA) of durable items, animals and agricultural land owned by households. The constructed PCA-based scores of households were divided into five quintiles and three wealth categories, where households’ residents with the lowest 40%, the middle 40% and the highest 20% of household wealth quintiles were classified as low, middle and high, respectively[25]. Categorical variables were presented in frequencies. The association between independent and dependent variables was tested using Pearson’s chi-square with reporting odds ratio (OR) and its corresponding 95% confidence interval (CI). Multivariable analysis using entry binary logistic regression model was conducted including all predicting variables and the adjusted OR with its corresponding 95%CI were reported. *P*-value of <0.05 was considered significant.

### Ethics statement

The study protocol was approved by Research and Ethics Committee (REC) of the Faculty of Medicine and Health Sciences, Sana’a University. Approval of school headmasters/ headmistresses was also taken after explaining the significance of the study. Each child was voluntary involved after receiving information in a way that the child can understand and give his/her assent. No informed consent was obtained from child’s parents/guardians, although they were informed about the study and had the right to refuse the participation of their child. Anonymity, dignity and privacy of the child and his/her family were protected.

## Results

### Characteristic of study population

Table 1 summarizes the characteristics of participants. A total of 445 schoolchildren were enrolled in this study. Their age ranged between 5 and 15 years with a mean of 10 ± 2.54. The majority of children (70.6%) were in the age group of 5–11 years. About 82% of the children belonged to families with more than 5 members. More than half of the children were living in houses without proper sanitation coverage (no toilet or flush/pour flush to open area) and about one-third had unimproved source of drinking water. Although no much difference in the characteristics of the study participants between the two districts, the majority of schoolchildren in Al-Haimah Al-Dakheliah District are living in houses without access to improved sanitation.

**Table 1:** Distribution of the study population by socio-demographic information, Sana’a Governorate, Yemen.

Characters	All children	Al-Haimah Al-Dakheliah (N = 227)	Bani Mater (N = 218)
	n (%)	n (%)	n (%)
**Gender**			
Male	230 (51.7)	128(56.4)	102(46.8)
Female	215 (48.3)	99(43.6)	116(53.2)
**Age (Years)**			
5–11	314 (70.6)	165(72.7)	149(68.3)
12–15	131 (29.4)	62(27.3)	69 (31.7)
**Father Education**			
Diploma and above	29 (6.5)	17(7.4)	12(5.5)
Secondary school	90 (20.2)	50(22.1)	40(18.4)
Primary school	176 (39.6)	86(37.9)	90(41.3)
Uneducated	150 (33.7)	74(32.6)	76(34.9)
**Mother Education**			
Diploma and above	5 (1.1)	0(0.0)	5(2.3)
Secondary school	17 (3.8)	3(1.3)	14(6.4)
Primary school	68 (15.3)	28 (12.3)	40(18.3)
Uneducated	355 (79.8)	196(86.3)	159(72.9)
**Household’s size**			
≤ 5 members	79 (17.8)	24(10.6)	55(25.2)
> 5 members	366 (82.2)	203(89.4)	163(74.8)
**Sanitation coverage**			
Flush/pour flush toilet to piped sewer system or Pit latrine	208 (46.7)	7(3.1)	201(92.2)
Flush/pour flush toilet to open area	169 (38.0)	153(67.4)	16(7.3)
No toilet	68 (15.3)	67(29.5)	1(0.5)
**Water coverage**			
Improved*	282 (63.4)	149(65.5)	133(61.0)
Unimproved^#^	163 (36.6)	78(34.4)	85(39.0)
**Wealth indices**			
Rich	89 (20.0)	23(10.1)	66(30.3)
Middle	178 (40.0)	113(49.8)	65(29.8)
Poor	178 (40.0)	91(40.1)	87(39.9)

*, Piped water into dwelling/yard, public tab

#, Dug well, Tanker-truck, Surface water.

### Prevalence and factors associated with *Schistosoma mansoni*

The prevalence of *S*. *mansoni* was higher in Al-Haimah Al-Dakheliah (33.9%) than in Bani Mater (1.4%). The intensity of *S*. *mansoni* was classified as heavy (4.1%), moderate (3.6%) and light intensity infection (10.3%) (Table 2).

**Table 2:** Distribution of *Schistosoma mansoni* infection among schoolchildren in the rural areas of Sana’a Governorate, Yemen (N = 445).

	Prevalence	
Type of infection	n (%)	95%CI
***S*. *mansoni* according to districts**		
Al-Haimah Al-Dakheliah (N = 227)	77(33.9)	(28.1, 40.3)
Bani Mater (N = 218)	3(1.4)	(0.5, 4.0)
**Intensity of *S*. *mansoni***		
Heavy intensity infection	18 (4.1)	(2.6, 6.3)
Moderate intensity infection	16 (3.6)	(2.3, 5.8)
Light intensity infection	46 (10.3)	(7.8, 13.5)

**N**, samples examined; **n**, samples positive for the infection; **CI**, Confidence interval

Univariable analysis was restricted to Al-Haimah Al-Dakheliah District where the prevalence of schistosomiasis was high, which identified a significant association between *S*. *mansoni* infection and uneducated mother (OR = 3.0, 95% CI: 1.11, 8.21, *P* = 0.024) and households without tap water (OR = 3.5, 95% CI: 1.49, 8.29, *P* = 0.003). Multivariate analysis identified households without tap water (adjusted OR = 2.9, 95% CI: 1.12, 7.55, *P* = 0.028) as an independent risk factor of S. *mansoni* (Table 3).

**Table 3:** Factors associated with *Schistosoma mansoni* among schoolchildren in Sana’a Governorate, Yemen (N = 227)^&^.

		*S*. *mansoni* infection			
Variable	N	n (%)	OR (95%CI)	AOR (95%CI)	P value
**Gender**					
Male	128	46 (35.9)	Reference		
Female	99	31 (31.3)	0.8(0.47, 1.42)	1.0(0.52, 1.98)	0.968
**Age (Years)**					
11–15	101	34(33.7)	Reference		
5–10	126	43(34.1)	1.0(0.59, 1.78)	1.1(0.59, 1.98)	0.794
**Household’s size**					
≤ 5 members	24	8(33.3)	Reference		
> 5 members	203	72(34.0)	1.0(0.42, 2.53)	1.4(0.55, 3.68)	0.470
**Father Education**					
Educated	153	50 (32.7)	Reference		
Uneducated	74	27 (36.5)	1.2(0.66, 2.12)	1.0(0.54, 1.93)	0.961
**Mother Education**					
Educated	31	5 (16.1)	Reference		
Uneducated	196	72 (36.7)	3.0(1.11, 8.21)	2.5(0.84, 7.23)	0.102
**Sanitation coverage** ^#^					
Improved sanitation	7	1 (14.3)	Reference		
Unimproved sanitation	237	76 (34.5)	3.2(0.37, 26.8)	2.2(0.24, 20, 87)	0.479
Source of drinking water*					
Tap water	46	7 (15.2)	Reference		
Other sources	181	70 (38.7)	3.5(1.49, 8.29)	2.9 (1.12, 7.55)	0.028
**Wealth indices**					
Rich	23	7(30.4)	Reference		
Middle	113	31(27.4)	0.9(0.32, 2.30)	0.7(0.25, 2.08)	0.540
Poor	91	39(42.9)	1.7(0.64, 4.57)	1.3(0.42, 3.78)	0.686
**Swimming in ponds or dams**					
No	69	20(29.0)	Reference		
Always/sometimes	158	57(36.1)	1.4(0.75, 2.55)	0.9(0.42, 1.99)	0.822

**N**, number of children examined; **n**, number of infected children; **OR**, Odds ratio; **AOR**; adjusted odds ratio **CI**, Confidence intervals

*, Other sources of drinking water (Dug well + Tanker-truck + Surface water)

**#,** Improved sanitation (Flush/pour flush toilet to piped sewer system or Pit latrine) and unimproved sanitation (no toilet or Flush/pour flush toilet to open area)

**&;** the analysis was restricted to Al-Haimah Al-Dakheliah District where the prevalence of schistosomiasis was high.

### Prevalence and factors associated with stunting, wasting and underweight

The prevalence of stunting among schoolchildren was 45.8% with 26.3% of the children being severely stunted while the prevalence of wasting was 25% with 9.7% of the children being severely wasted. Children aged 11–15 years had significantly higher rates of stunting and wasting than children aged 5–10 years. Among schoolchildren aged 5–10 years, 27.3% of them were diagnosed as underweight and 10.8% were classified as severe underweight (Table 4).

**Table 4:** Prevalence of underweight, stunting and wasting among schoolchildren in Sana’a Governorate, Yemen*.

Variable	5–10 years (N = 231)		11–15 years (N = 214)		5–15 years (N = 445)	
	n (%)	95%CI	n (%)	95%CI	n (%)	95%CI
**Underweight** *						
Moderate/Severe (WAZ < - 2SD)	63 (27.3)	(21.9, 33.4)	NA	NA	NA	NA
Moderate (WAZ = - 3SD to -2SD)	38 (16.5)	(12.2, 21.8)	NA	NA	NA	NA
Severe (WAZ < - 3SD)	25 (10.8)	(7.4, 15.5)	NA	NA	NA	NA
**Stunting**						
Moderate/Severe (HAZ < - 2SD)	61 (26.4)	(20.8, 32.6)	143(66.9)	(60.1, 73.1)	204(45.8)	(41.2, 50.5)
Moderate (HAZ = - 3SD to -2SD)	40 (17.3)	(12.7, 22.8)	47 (22.0)	(16.6, 28.1)	87 (19.6)	(16.1, 23.5)
Severe (HAZ < - 3SD)	21 (9.1)	(5.7, 13.6)	96 (44.9)	(38.1, 51.8)	117(26.3)	(22.4, 30.6)
**Wasting**						
Moderate/Severe (BAZ < - 2SD)	36 (15.6)	(11.2, 20.9)	75 (35.1)	(28.7, 41.8)	111(25.0)	(21.2, 29.2)
Moderate (BAZ = - 3SD to -2SD)	24 (10.4)	(12.2, 18.9)	44 (20.6)	(15.4, 26.6)	68 (15.3)	(12.2, 18.9)
Severe (BAZ < - 3SD)	12 (5.2)	(6.7, 15.1)	31 (14.5)	(10.1, 19.9)	43 (9.7)	(7.3, 12.8)

**N**; number of children enrolled in the study, **n**; number of malnourished children, **CI**; confidence interval, **NA**; not applicable, **WAZ**; Weight-for-Age Z-score, **HAZ**; Height-for-age Z-score, **BAZ**; BMI-for-Age Z-score

*; The WHO reference data for WAZ used by the WHO AnthroPlus software were for age ≤ 10 years, therefore underweight was estimated for children aged 5–10 years (n = 231)

Univariable analysis showed that schoolchildren resident in Al-Haimah Al -Dakheliah District (OR = 2.2, 95%CI:1.20, 4.06, *P* = 0.011) and living in houses with unimproved sanitation (OR = 1.9, 95%CI:1.02, 3.41, *P* = 0.043) or infected with *S*. *mansoni* (OR = 2.0, 95%CI: 1.01, 4.05, *P* = 0.045) were at high risk of developing underweight. However, underweight had negative association with educated fathers (OR = 0.5, 95%CI: 0.27, 0.98, *P* = 0.041). Multivariable analysis did not identify an independent risk factor of underweight (Table 5).

**Table 5:** Factors associated with underweight among schoolchildren, Sana’a Governorate, Yemen (N = 231).

Variable		Underweight			
	N	*n*(%)	OR (95%CI)	AOR (95%CI)	*P value*
**Gender**					
Male	124	34 (27.4)	Reference		
Female	107	29 (27.1)	1.0 (0.55, 1.76)	1.0(0.53, 1.85)	0.966
**District**					
Bani mater	105	20(19.0)	Reference		
Al-Haimah Al - Dakheliah	126	43(34.1)	2.2 (1.20, 4.06)	4.7(0.85, 26.29)	0.077
**Household’s size**					
≤ 5 members	46	13 (28.3)	Reference		
>5 members	185	50 (27.0)	0.9 (0.46, 1.93)	0.8(0.37, 1.72)	0.561
**Father Education**					
Educated	148	47(31.8)	Reference		
Uneducated	83	16(19.3)	0.5 (0.27, 0.98)	0.5(0.25, 1.07)	0.077
**Mother Education**					
Educated	50	17 (34.0)	Reference		
Uneducated	181	46 (25.4)	0.7 (0.34, 1.30)	0.6(0.27, 1.38)	0.238
**Sanitation coverage** ^#^					
Improved sanitation	102	21(20.6)	Reference		
Unimproved sanitation	129	42(32.6)	1.9 (1.02, 3.41)	0.5(0.08, 2.35)	0.340
Source of drinking water*					
Tap water	75	18 (24.0)	Reference		
Other sources	156	45 (28.8)	1.3 (0.68, 2.42)	1.0(0.49, 2.06)	0.992
**Wealth indices**					
Rich	41	9 (22.0)	Reference		
Middle	71	19 (26.8)	1.3 (0.52, 3.22)	0.9(0.31, 2.38)	0.764
Poor	119	35 (29.4)	1.5 (0.64, 3.43)	1.3(0.52, 3.34)	0.652
***S*. *mansoni***					
Not infected	188	46 (24.5)	Reference		
Infected	43	17 (39.5)	2.0 (1.01, 4.05)	1.5(0.67, 3.53)	0.309
***E*. *histolytica***					
Not infected	133	39 (29.3)	Reference		
Infected	98	24 (24.5)	0.8 (0.43, 1.41)	0.9(0.46, 1.61)	0.637
***G*. *lamblia***					
Not infected	181	46 (25.4)	Reference		
Infected	50	17 (34.0)	1.5 (0.77, 2.67)	1.5(0.71, 3.06)	0.294

**N**, number of children examined; ***n***, number of malnourished children; **OR**, Odds ratio; **AOR**, Adjusted odds ratio; **CI**, Confidence intervals

*Other sources of drinking water (Dug well + Tanker-truck + Surface water)

^**#**^, Improved (Flush/pour flush toilet to piped sewer system or Pit latrine) and Unimproved (no toilet or Flush/pour flush toilet to open area)

^***&***^, underweight was measured for children aged 5–10 years.

Stunting was significantly associated with children aged 11–15 years (OR = 5.6, 95%CI: 3.73, 8.44, *P* <0.001), females (OR = 1.5, 95%CI: 1.01, 2.13, *P* = 0.047) and *S*. *mansoni* infection (OR = 4.0, 95%CI: 2.00, 8.01, *P* = 0.002). Multivariable analysis identified the infection with *S*. *mansoni* and early adolescence as independent risk factors of stunting (Table 6).

**Table 6:** Factors associated with stunting among schoolchildren, Sana’a Governorate, Yemen (N = 445).

		Stunted children			
Variable	N	*n*(%)	OR (95%CI)	AOR (95%CI)	*P value*
**Age (Years)**					
5–10	231	61(26.4)	Reference		
11–15	214	143(66.8)	5.6(3.73, 8.44)	6.8(4.26, 10.82)	< 0.001
**Gender**					
Male	230	95 (41.3)	Reference		
Female	215	109 (50.7)	1.5(1.01, 2.13)	1.4(0.90, 2.13)	0.144
**District**					
Bani mater	218	107(49.1)	Reference		
Al-Haimah Al - Dakheliah	227	97(42.7)	0.8(0.53, 1.13)	0.4(0.13, 1.08)	0.070
**Household’s size**					
≤ 5 members	79	40(50.6)	Reference		
>5 members	366	164(44.8)	0.8(0.49, 1.29)	0.7(0.38, 1.21)	0.187
**Father Education**					
Educated	295	145 (49.2)	Reference		
Uneducated	150	59 (39.3)	0.7(0.45, 1.00)	0.7(0.40, 1.06)	0.083
**Mother Education**					
Educated	90	43(47.8)	Reference		
Uneducated	355	161(45.4)	0.9(0.57, 1.44)	0.9(0.50, 1.60)	0.698
**Sanitation coverage** ^#^					
Improved sanitation	208	99 (47.6)	Reference		
Unimproved sanitation	237	105 (44.3)	0.9(0.60, 1.27)	1.5(0.55, 4.20)	0.418
Source of drinking water*					
Tap water	113	44 (38.9)	Reference		
Other sources	332	160 (48.2)	1.5(0.94, 2.25)	0.8(0.49, 1.44)	0.529
**Wealth indices**					
Rich	89	42(47.2)	Reference		
Middle	178	87(48.9)	1.1(0.64, 1.78)	1.2(0.64, 2.19)	0.600
Poor	178	75(42.1)	0.8(0.49, 1.36)	1.3(0.71, 2.50)	0.379
***S*. *mansoni***					
Not infected	365	155(42.5)	Reference		
Infected	80	49 (61.3)	4.0(2.00, 8.01)	4.1(2.16, 7.84)	< 0.001
***E*. *histolytica***					
Not infected	250	120 (48.0)	Reference		
Infected	195	84 (43.1)	0.8(0.56, 1.20)	0.8(0.50, 1.19)	0.242
***G*. *lamblia***					
Not infected	355	160 (45.1)	Reference		
Infected	90	44 (48.9)	1.2(0.73, 1.85)	1.5(0.87, 2.55)	0.148

**N**, number of children examined; ***n***, number of malnourished children; ***P***, p value; **OR**, Odds ratio; **AOR**, Adjusted odds ratio; **CI**, Confidence intervals

*Other sources of drinking water (Dug well + Tanker-truck + Surface water)

^**#**^, Improved (Flush/pour flush toilet to piped sewer system or Pit latrine) and Unimproved (no toilet or Flush/pour flush toilet to open area).

Wasting was associated with children aged 11–15 years (OR = 2.9, 95%CI: 1.86, 4.60, *P* < 0.001), households without tap water (OR = 1.9, 95%CI: 10, 3.28, *P* = 0.021) and children from families placed in the middle category of wealth indices (OR = 2.2, 95%CI: 1.12, 4.15, *P* = 0.022). Multivariable analysis identified children aged 11–15 years, and the middle and poor categories of wealth indices as independent risk factors of wasting (Table 7).

**Table 7:** Factors associated with wasting among schoolchildren, Sana’a Governorate, Yemen (N = 445).

		Wasted children			
Variable	N	*n*(%)	OR (95%CI)	AOR (95%CI)	*P value*
**Age (Years)**					
5–10	231	36(15.6)	Reference		
11–15	214	75(35.0)	2.9(1.86, 4.60)	3.1(1.86, 4.97)	<0.001
**Gender**					
Male	230	56 (24.3)	Reference		
Female	215	55 (25.6)	1.1(0.70, 1.64)	1.0(0.64, 1.60)	0.947
**District**					
Bani mater	218	53(24.3)	Reference		
Al-Haimah Al -Dakheliah	227	58(25.6)	1.1(0.70, 1.64)	0.9(0.31, 2.78)	0.898
**Household’s size**					
≤ 5 members	79	22(27.8)	Reference		
>5 members	366	89(24.3)	0.8(0.48, 1.44)	0.8(0.42, 1.40)	0.389
**Father Education**					
Educated	295	75 (25.4)	Reference		
Uneducated	150	36 (24.0)	0.9(0.59, 1.46)	1.0(0.57, 1.58)	0.844
**Mother Education**					
Educated	90	19(21.1)	Reference		
Uneducated	355	92(25.9)	1.3(0.75, 2.29)	1.0(0.54, 1.93)	0.954
**Sanitation coverage** ^#^					
Improved sanitation	208	50 (24.0)	Reference		
Unimproved sanitation	237	61 (25.7)	1.1(0.71, 1.69)	1.2(0.41, 3.30)	0.783
Source of drinking water*					
Tap water	113	19 (16.8)	Reference		
Other sources	156	92 (27.7)	1.9(1.10, 3.28)	1.4(0.77, 2.55)	0.270
**Wealth indices**					
Rich	89	14(15.7)	Reference		
Middle	178	51(28.7)	2.2(1.12, 4.15)	2.1(1.01, 4.15)	0.046
Poor	178	46(25.8)	1.9(0.96, 3.62)	2.3(1.11, 4.77)	0.026
***S*. *mansoni***					
Not infected	365	90(24.7)	Reference		
Infected	80	21 (26.3)	1.1(0.63, 1.89)	1.0(0.51, 1.85)	0.923
***E*. *histolytica***					
Not infected	250	65 (26.0)	Reference		
Infected	195	46 (23.6)	0.9(0.57, 1.36)	0.9(0.54, 1.35)	0.504
***G*. *lamblia***					
Not infected	355	93 (26.2)	Reference		
Infected	90	18 (20.0)	0.7(0.40, 1.24)	0.7(0.39, 1.30)	0.267

**N**, number of children examined; ***n***, number of malnourished children; ***P***, p value; **OR**, Odds ratio; **AOR**, Adjusted odds ratio; **CI**, Confidence intervals

*Other sources of drinking water (Dug well + Tanker-truck + Surface water)

^**#**^, Improved (Flush/pour flush toilet to piped sewer system or Pit latrine) and Unimproved (no toilet or Flush/pour flush toilet to open area).

### Prevalence and factors associated with anemia

The prevalence of anemia among schoolchildren was 31.7% (95%CI: 27.5%, 36.2%) with 0.5%, 21.1% and 10.1% being severe, moderate and mild anemia, respectively. Univariable analysis showed that schoolchildren from Al-Haimah Al–Dakheliah District (OR = 3.9, 95%CI: 2.12, 6.97, *P* <0.001), whose mothers were uneducated (OR = 2.5, 95%CI: 1.11, 5.69, *P* = 0.023), and those living in houses with unimproved sanitation (OR = 3.2, 95%CI: 1.11, 5.69, *P* <0.001) and without tap water (OR = 2.2, 95%CI: 1.10, 4.52, *P* = 0.024) were at higher risk of anemia. Multivariable analysis using binary logistic regression model identified female (AOR = 1.6, 95%CI:1.03, 2.43, *P* = 0.038), Al-Haimah Al–Dakheliah District (AOR = 3.4, 95%CI:1.20, 9.55, *P* = 0.021) and early adolescence (11–15 years) (AOR = 1.8, 95%CI:1.14, 2.78, *P* = 0.011) as independent risk factors of anemia among schoolchildren (Table 8).

**Table 8:** Factors associated with anemia schoolchildren in Sana’a Governorate, Yemen (N = 445).

Variable		Anemia			
	N	n (%)	OR (95%CI)	AOR (95%CI)	P value
**Gender**					
Male	230	64 (27.8)	Reference		
Female	215	77 (35.8)	1.5(0.96, 2.2)	1.6(1.03, 2.43)	0.038
**Age (Years)**					
5–10	231	62(26.8)	Reference		
11–15	214	79(36.9)	1.6(1.1, 2.5)	1.8(1.14, 2.78)	0.011
**District**					
Bani mater	218	48(22.0)	Reference		
Al-Haimah Al - Dakheliah	227	93(41.0)	2.5 (1.6, 3.7)	3.4(1.20, 9.55)	0.021
**Household’s size**					
≤ 5 members	79	22(27.8)	Reference		
> 5 members	366	119(32.5)	1.3 (0.73. 2.1)	1.0(0.56, 1.81)	0.976
**Father Education**					
Educated	295	102 (34.6)	Reference		
Uneducated	150	39 (26.0)	0.7(0.44, 1.1`)	0.7(0.45, 1.17)	0.188
**Mother Education**					
Educated	90	27 (30.0)	Reference		
Uneducated	355	114 (32.1)	1.1(0.7, 1.8)	1.0(0.53, 1.70)	0.860
**Sanitation coverage** ^**#**^					
Improved sanitation	208	46 (22.1)	Reference		
Unimproved sanitation	237	95 (40.1)	2.4 (1.6, 3.6)	1.0(0.37, 2.64)	0.975
**Source of drinking water** *					
Tap water	113	25 (22.1)	Reference		
Other sources	332	116 (34.9)	1.9 (1.2, 3.1)	1.6(0.94, 2.85)	0.084
**Wealth indices**					
Rich	89	25(28.1)	Reference		
Middle	178	56(31.5)	1.1(0.7, 2.0)	0.7(0.40, 1.39)	0.347
Poor	178	60 (33.7)	1.2(0.7, 2.0)	1.1(0.61, 2.15)	0.677
***S*. *mansoni***					
No	365	113(31.0)	Reference		
Yes	80	28 (35.0)	1.2(0.7, 2.1)	0.6(0.36, 1.14)	0.131
***E*. *histolytica***					
No	250	78 (31.2)	Reference		
Yes	195	63(32.3)	1.1(0.7, 1.6)	1.1(0.73, 1.72)	0.603
***G*. *lamblia***					
No	355	111 (31.3)	Reference		
Yes	90	30 (33.3)	1.1(0.7, 1.8)	1.0(0.60, 1.71)	0.970

**N**, number of children examined; **n**, number of malnourished children; **OR**, Odds ratio; **AOR**, adjusted odds ratio**; CI**, Confidence intervals

*, Other sources of drinking water (Dug well + Tanker-truck + Surface water)

**#,** Improved sanitation (Flush/pour flush toilet to piped sewer system or Pit latrine) and unimproved sanitation (no toilet or Flush/pour flush toilet to open area).

## Discussion

The present study indicated focal prevalence of *S*. *mansoni* among schoolchildren in Sana’a Governorate. At district level, the study placed Al-Haimah Al–Dakheliah and Bani Mater districts at high and low risk of schistosomiasis (33.9% and 1.4%, respectively). The presence of foci with high infection rates, although the pressure of MDA campaigns, can be explained by the high reinfection rate of *S*. *mansoni*. A recent study conducted in Ethiopia reported high reinfection rate of *S*. *mansoni* after 6 months of treatment with praziquantel[26]. The reinfection with *S*. *mansoni* was found to be affected by socioeconomic status, level of education of the household head and the baseline heavy infection[27], which may justify the variation in the prevalence of *S*. *mansoni* between the two districts. These findings, in turn, suggest that MDA campaigns should be integrated with additional measures to control schistosomiasis.

Multivariable analysis identified having no tap water at home as an independent risk factor of *S*. *mansoni* in Al-Haimah Al–Dakheliah District. This observation could be explained by the possibility of children’s responsibility of bringing household’s water, a common practice in Yemen, which increased their contact with unsafe water and made them prone to *S*. *mansoni* infection [28,29]. The result suggests an integration of MDA of praziquantel and the delivery of a community-based WASH programme as an effective approach for combating schistosomiasis in these communities. The positive impact of WASH intervention on deworming programmes has been well evidenced [30,31].

The nutritional status of schoolchildren in Yemen has been neglected despite its significant impact on cognitive and educational achievement[5]. In the present study, the prevalence of stunting, underweight and wasting among schoolchildren aged 5–15 years was 45.8%, 27.3% and 25%, respectively. The study reported lower prevalence of stunting and underweight and five-times higher prevalence of wasting compared to stunting, underweight and wasting reported in previous studies conducted among schoolchildren in Al Mahweet and Sada’ah governorates, Yemen[19,20]. The prevalence of stunting and wasting were higher among schoolchildren aged 11–15 years than those aged 5–10 years. These findings are consistent with previous studies conducted in Pakistan [32], Tanzania[33] and Madagascar[34]. The increased prevalence of stunting and wasting with age could be explained in part by the accumulated exposure of the older children to childhood diseases and inadequate diets [35].

*Schistosoma mansoni* is an independent risk factor of stunting among schoolchildren in Sana’a Governorate. The association between *S*. *mansoni* and stunting was reported in different studies [4,36,37].

Underweight takes into account both acute malnutrition (wasting) and chronic malnutrition (stunting). In the present study, a significant association between *S*. *mansoni* and underweight was found using univariable analysis although the multivariable analysis model did not confirm this association. Schoolchildren belonging to families with poor and middle wealth indices were at high risk of being acute malnourished. This finding is consistent with previous reports from Ethiopia [38] and India [39], which could reflect the inadequate feeding among children from families with low wealth indices.

The prevalence of anemia represents a moderate and severe public health problem among schoolchildren in Bani Matar and Al-Haimah Al -Dakheliah districts, respectively. Although the causes of anemia in the present study have not been identified, iron deficiency is one of the primary causes of anemia in the Yemeni communities [40]. The reason behind the high prevalence of anemia in Al-Haimah Al -Dakheliah District is not clear, although it may be attributed to socioeconomic status: 90% of the children belonged to families with moderate and poor wealth indices. Schoolchildren aged 11–15 years were at two times higher risk of being anemic compared to younger age group. This finding is consistent with previous studies conducted among children in different countries [41–46], which could be explained by the hyperactivity during this age together with high demand of micronutrient and limited consumption of a variety of food sources due to the household food insecurity [47], which can be reduced by school feeding [48,49]. The gender female was also an independent risk factor of anemia, which is in line with previous studies [50,51]. It is noteworthy that anemia among schoolchildren may lead to impaired cognitive function [52]. No significant association was found between *S*. *mansoni* and anemia, which is consistent with previous studies conducted elsewhere [28,33].

The present study is limited by the low number of districts and schools enrolled in the study, which prevents the conclusion about the overall prevalence of schistosomiasis at governorate level because the disease is highly focal, although study findings are consistent with the results of the latest nationwide survey. However, the study sample size and design are appropriate to assess the association between schistosomiasis and nutritional status of schoolchildren in Sana’a Governorate.

In conclusion, the study findings showed highly focal prevalence of schistosomiasis in Sana’a Governorate with a public health significance that varies from low to high risk. Schoolchildren living in houses without tap water are at high risk of the infection. Schoolchildren harboring the parasite and early adolescent children had high prevalence of stunting. Besides, early adolescent schoolchildren and children belonged to families with middle or poor wealth were wasted. Anemia is a moderate public health threat with early adolescent and female schoolchildren, being at higher risk. The study findings suggest adopting integrated control measures for the control of schistosomiasis such as MDA and WASH, and integrated diseases control progarmmes for improving the health status of schoolchildren.

## References

[1] World Health Organization. 2018. Schistosomiasis: status of schistosomiasis endemic countries World Health Organization; [Available from: https://apps.who.int/neglected_diseases/ntddata/sch/sch.html.

[2] GBD DALYs and Hale Collaborators. 2018. Global, regional, and national disability-adjusted life-years (DALYs) for 359 diseases and injuries and healthy life expectancy (HALE) for 195 countries and territories, 1990–2017: a systematic analysis for the Global Burden of Disease Study 2017. Lancet. 392(10159):1859–922. doi: 10.1016/S0140-6736(18)32335-3 30415748 PMC6252083

[3] Koukounari A, Fenwick A, Whawell S, Kabatereine NB, Kazibwe F, Tukahebwa EM, et al. 2006. Morbidity indicators of Schistosoma mansoni: relationship between infection and anemia in Ugandan schoolchildren before and after praziquantel and albendazole chemotherapy. The American journal of tropical medicine and hygiene. 75(2):278–86. 16896133

[4] Kabongo MM, Linsuke S, Baloji S, Mukunda F, Raquel IDL, Stauber C, et al. 2018. Schistosoma mansoni infection and its association with nutrition and health outcomes: a household survey in school-aged children living in Kasansa, Democratic Republic of the Congo. The Pan African medical journal. 31:197. doi: 10.11604/pamj.2018.31.197.16364 31086641 PMC6488962

[5] Ezeamama AE, Bustinduy AL, Nkwata AK, Martinez L, Pabalan N, Boivin MJ, et al. 2018. Cognitive deficits and educational loss in children with schistosome infection-A systematic review and meta-analysis. PLoS neglected tropical diseases. 12(1):e0005524. doi: 10.1371/journal.pntd.0005524 29329293 PMC5766129

[6] Grevai S. 1922. Schistosomiasis (Bilharziasis) in Arabia. Ind J Med Research 10:943–7.

[7] Kuntz RE. 1952. Schistosoma mansoni and Shaematobium in the Yemen, Southwest Arabia; with a report of an unusual factor in the epidemiology of Schistosomiasis mansoni. J Parasitol. 38(1):24–8. 14928148

[8] Kuntz RE, Malakatis GM, Lawless DK, Strome CP. 1953. Medical mission to the Yemen, Southwest Arabia, 1951. II. A cursory survey of the intestinal protozoa and helminth parasites in the people of the Yemen. The American journal of tropical medicine and hygiene. 2(1):13–9. doi: 10.4269/ajtmh.1953.2.13 13007917

[9] Kuz’mtsn IL. 1966. [Distribution and clinical features of urinary schistosomiasis in Yemen]. Med Parazitol (Mosk). 35(5):564–6. 6003345

[10] Parrinello A. 1966. [Electrocardiographic modifications from antimony in a group of Yemenite patients affected by schistosomiasis]. Rass Clin Ter. 65(2):121–5. 5943145

[11] Mancioli M, Parrinello A. 1967. [Nitrothiamidazole in the treatment of schistosomiasis in Yemen. Preliminary clinical study in relation to the possibility of a mass therapy]. Clin Ter. 42(1):15–55. 5597000

[12] Prokhorov AF. 1969. [On the problem of schistosomiasis in the Yemen Arab Republic]. Med Parazitol (Mosk). 38(1):91–4. 5371118

[13] Zaiachkovskii SM. 1971. [Epidemiology and clinical picture of intestinal schistosomiasis in Yemen]. Med Parazitol (Mosk). 40(4):409–11. 5134371

[14] Arfaa F. 1972. Studies on schistosomiasis in the Yemen Arab Republic. The American journal of tropical medicine and hygiene. 21(4):421–4. doi: 10.4269/ajtmh.1972.21.421 5065694

[15] Daffalla A. 1988. Schistosomiasis control project in Y.A.R. Assignment Report. WHO office, Sana’a, Yemen.

[16] Arfaa F. 1980. Schistosomiasis control: Southern Uplands Rural Development Project. Y.A.R. Assignment Report WHO office, Sana’a, Yemen.

[17] Johari NA. 2014. Mapping of Schistosomiasis and Soil-transmitted helminths in Yemen, and the push for elimination (master thesis). London: Imperial College.

[18] World Bank. 2018. Final Report of Schistosomiasis Control Project in Yemen (P113102). World Bank.

[19] Raja’a YA, Sulaiman SM, Elkarib SA, Mubarak JS. 2001. Nutritional status of Yemeni schoolchildren in Al-Mahweet Governorate. Eastern Mediterranean health journal 7(1–2):204–10. 12596971

[20] Raja’a YA, Mubarak JS. 2006. Intestinal parasitosis and nutritional status in schoolchildren of Sahar district, Yemen. Eastern Mediterranean Health Journal. 12. 17361690

[21] Katz N, Chaves A, Pellegrino J. 1972. A simple device for quantitative stool thick-smear technique in schistosomiasis mansoni. Rev Inst Med Trop Sao Paulo. 14(6):397–400. 4675644

[22] Montresor A, Crompton, David WT, Hall A, Bundy DAP, Savioli L, et al. 1998. Guidelines for the evaluation of soil-transmitted helminthiasis and schistosomiasis at community level: a guide for managers of control programmes World Health Organization.

[23] World Health Oganization. 2017. Haemoglobin concentrations for the diagnosis of anaemia and assessment of severity. Available from: http://www.who.int/vmnis/indicators/haemoglobin. pdf.

[24] World Health Oganization. 2009. WHO AnthroPlus for personal computers Manual: Software for assessing growth of the world’s children and adolescents. Geneva: World Health Organization.

[25] Vyas S, Kumaranayake L. 2006. Constructing socio-economic status indices: how to use principal components analysis. Health policy and planning. 21(6):459–68. doi: 10.1093/heapol/czl029 17030551

[26] Woldegerima E, Bayih AG, Tegegne Y, Aemero M, Jejaw Zeleke A. 2019. Prevalence and Reinfection Rates of Schistosoma mansoni and Praziquantel Efficacy against the Parasite among Primary School Children in Sanja Town, Northwest Ethiopia. Journal of parasitology research. 2019:3697216. doi: 10.1155/2019/3697216 31179124 PMC6507171

[27] Gazzinelli A, Oliveira-Prado R, Matoso LF, Veloso BM, Andrade G, Kloos H, et al. 2017. Schistosoma mansoni reinfection: Analysis of risk factors by classification and regression tree (CART) modeling. PloS one. 12(8):e0182197. doi: 10.1371/journal.pone.0182197 28813451 PMC5558968

[28] Tukahebwa EM, Magnussen P, Madsen H, Kabatereine NB, Nuwaha F, Wilson S, et al. 2013. A very high infection intensity of Schistosoma mansoni in a Ugandan Lake Victoria Fishing Community is required for association with highly prevalent organ related morbidity. PLoS neglected tropical diseases. 7(7):e2268. doi: 10.1371/journal.pntd.0002268 23936559 PMC3723538

[29] Matthys B, Tschannen AB, Tian-Bi NT, Comoe H, Diabate S, Traore M, et al. 2007. Risk factors for Schistosoma mansoni and hookworm in urban farming communities in western Cote d’Ivoire. Tropical medicine & international health: TM & IH. 12(6):709–23. doi: 10.1111/j.1365-3156.2007.01841.x 17550468

[30] Vaz Nery S, Pickering AJ, Abate E, Asmare A, Barrett L, Benjamin-Chung J, et al. 2019. The role of water, sanitation and hygiene interventions in reducing soil-transmitted helminths: interpreting the evidence and identifying next steps. Parasites & vectors. 12(1):273. doi: 10.1186/s13071-019-3532-6 31138266 PMC6540378

[31] Vaz Nery S, Traub RJ, McCarthy JS, Clarke NE, Amaral S, Llewellyn S, et al. 2019. WASH for WORMS: A Cluster-Randomized Controlled Trial of the Impact of a Community Integrated Water, Sanitation, and Hygiene and Deworming Intervention on Soil-Transmitted Helminth Infections. The American journal of tropical medicine and hygiene. 100(3):750–61. doi: 10.4269/ajtmh.18-0705 30628573 PMC6402916

[32] Khuwaja S, Selwyn BJ, Shah SM. 2005. Prevalence and correlates of stunting among primary school children in rural areas of southern Pakistan. Journal of tropical pediatrics. 51(2):72–7. doi: 10.1093/tropej/fmh067 15677373

[33] Munisi DZ, Buza J, Mpolya EA, Kinung’hi SM. 2016. Schistosoma mansoni Infections, Undernutrition and Anaemia among Primary Schoolchildren in Two Onshore Villages in Rorya District, North-Western Tanzania. PloS one. 11(12):e0167122. doi: 10.1371/journal.pone.0167122 27936031 PMC5147845

[34] Aiga H, Abe K, Andrianome VN, Randriamampionona E, Razafinombana AR, Murai T, et al. 2019. Risk factors for malnutrition among school-aged children: a cross-sectional study in rural Madagascar. BMC public health. 19(1):773. doi: 10.1186/s12889-019-7013-9 31208397 PMC6580631

[35] Dureab F, Al-Falahi E, Ismail O, Al-Marhali L, Al Jawaldeh A, Nuri NN, et al. 2019. An Overview on Acute Malnutrition and Food Insecurity among Children during the Conflict in Yemen. Children (Basel). 6(6). doi: 10.3390/children6060077 31195654 PMC6616580

[36] Sircar AD, Mwinzi PNM, Onkanga IO, Wiegand RE, Montgomery SP, Secor WE. 2018. Schistosoma mansoni Mass Drug Administration Regimens and Their Effect on Morbidity among Schoolchildren over a 5-Year Period-Kenya, 2010–2015. The American journal of tropical medicine and hygiene. 99(2):362–9. doi: 10.4269/ajtmh.18-0067 29893197 PMC6090338

[37] Assis AM, Prado MS, Barreto ML, Reis MG, Conceicao Pinheiro SM, Parraga IM, et al. 2004. Childhood stunting in Northeast Brazil: the role of Schistosoma mansoni infection and inadequate dietary intake. Eur J Clin Nutr. 58(7):1022–9. doi: 10.1038/sj.ejcn.1601926 15220944

[38] Getaneh Z, Melku M, Geta M, Melak T, Hunegnaw MT. 2019. Prevalence and determinants of stunting and wasting among public primary school children in Gondar town, northwest, Ethiopia. BMC pediatrics. 19(1):207. doi: 10.1186/s12887-019-1572-x 31238889 PMC6591879

[39] Tigga PL, Sen J, Mondal N. 2015. Association of some socio-economic and socio-demographic variables with wasting among pre-school children of North Bengal, India. Ethiop J Health Sci. 25(1):63–72. doi: 10.4314/ejhs.v25i1.9 25733786 PMC4337084

[40] Ministry of Public Health and Population. 2013. Yemen National Health and Demographic Survey. Ministry of Health and Population, Yemen.

[41] Chandrakumari AS, Sinha P, Singaravelu S, Jaikumar S. 2019. Prevalence of Anemia Among Adolescent Girls in a Rural Area of Tamil Nadu, India. Journal of family medicine and primary care. 8(4):1414–7. doi: 10.4103/jfmpc.jfmpc_140_19 31143731 PMC6510068

[42] Elayappen A, Muthukumar A. 2014. Anemia in an Adolescent Male: Is It a Red Flag? Glob Pediatr Health. 1:2333794X14550524. 10.1177/2333794X14550524 PMC4804671 27335904

[43] Engidaw MT, Wassie MM, Teferra AS. 2018. Anemia and associated factors among adolescent girls living in Aw-Barre refugee camp, Somali regional state, Southeast Ethiopia. PloS one. 13(10):e0205381. doi: 10.1371/journal.pone.0205381 30308060 PMC6181359

[44] Rati SA, Jawadagi S. 2014. Prevalence of Anemia among adolescent girls studying in selected schools. Int J Sci Res. 3(8):1237–42.

[45] Regasa RT, Haidar JA. 2019. Anemia and its determinant of in-school adolescent girls from rural Ethiopia: a school based cross-sectional study. BMC Womens Health. 19(1):98. doi: 10.1186/s12905-019-0791-5 31315626 PMC6637513

[46] Gonete KA, Tariku A, Wami SD, Derso T. 2018. Prevalence and associated factors of anemia among adolescent girls attending high schools in Dembia District, Northwest Ethiopia, 2017. Arch Public Health. 76:79. doi: 10.1186/s13690-018-0324-y 30598822 PMC6302287

[47] Adelman S, Gilligan DO, Konde-Lule J, Alderman H. 2019. School Feeding Reduces Anemia Prevalence in Adolescent Girls and Other Vulnerable Household Members in a Cluster Randomized Controlled Trial in Uganda. J Nutr. 149(4):659–66. doi: 10.1093/jn/nxy305 30926996 PMC6461720

[48] Neervoort F, von Rosenstiel I, Bongers K, Demetriades M, Shacola M, Wolffers I. et.al 2013. Effect of a school feeding programme on nutritional status and anaemia in an urban slum: a preliminary evaluation in Kenya. J Trop Pediatr. 59(3):165–74. doi: 10.1093/tropej/fms070 23243080

[49] Rakesh PS, George LS, Joy TM, George S, Renjini BA, Beena KV. et.al 2019. Anemia Among School Children in Ernakulam District, Kerala, India. Indian J Hematol Blood Transfus. 35(1):114–8. doi: 10.1007/s12288-018-1001-6 30828157 PMC6369096

[50] Abou-Zeid AH, Abdel-Fattah MM, Al-Shehri AS, Hifnawy TM, Al-Hassan SA. 2006. Anemia and nutritional status of schoolchildren living at Saudi high altitude area. Saudi Med J. 27(6):862–9. 16758052

[51] Egbi G, Steiner-Asiedu M, Kwesi FS, Ayi I, Ofosu W, Setorglo J, et al. Anaemia among school children older than five years in the Volta Region of Ghana. The Pan African medical journal. 2014;17 Suppl 1:10. doi: 10.11694/pamj.supp.2014.17.1.3205 24644526 PMC3948363

[52] Bahrami A, Khorasanchi Z, Tayefi M, Avan A, Seifi N, Tavakoly Sany SB, et al. 2019. Anemia is associated with cognitive impairment in adolescent girls: A cross-sectional survey. Appl Neuropsychol Child. 1–7. doi: 10.1080/21622965.2018.1550405 30661397

